# Infective endocarditis involving MitraClip^©^ devices: a systematic literature review

**DOI:** 10.1007/s15010-023-02067-y

**Published:** 2023-06-29

**Authors:** Lorenzo Bertolino, Mohammad Said Ramadan, Rosa Zampino, Emanuele Durante-Mangoni

**Affiliations:** 1 Department of Advanced Medical & Surgical Sciences, University of Campania ‘L. Vanvitelli’, Naples, Italy; 2 Unit of Infectious and Transplant Medicine, AORN Ospedali dei Colli-Monaldi Hospital, Piazzale Ettore Ruggieri, 80131 Naples, Italy; 3 Department of Precision Medicine, University of Campania ‘L. Vanvitelli’, Naples, Italy

**Keywords:** MitraClip, Infective endocarditis, Cardiac devices, Systematic review

## Abstract

**Purpose:**

Progress of interventional cardiology has boosted the use of newer cardiac devices. These devices are perceived to be less prone to infections compared to traditional surgical prostheses, but little data are currently available. In this systematic review (SR), we summarize current literature regarding the clinical characteristics, management, and outcomes of patients with MitraClip-related infective endocarditis (IE).

**Methods:**

We conducted a SR of PubMed, Google Scholar, Embase, and Scopus between January 2003 and March 2022. MitraClip-related IE was defined according to 2015 ESC criteria whereas MitraClip involvement as vegetation on the device or on the mitral valve. Risk of bias was assessed through standardized checklist and potential bias of underestimation cannot be excluded. Data regarding clinical presentation, echocardiography, management, and outcome were collected.

**Results:**

Twenty-six cases of MitraClip-related IE were retrieved. The median age of patients was 76 [61–83] years with a median EuroScore of 41%. Fever was present in 65.8% of patients followed by signs and symptoms of heart failure (42.3%). IE occurred early in 20 (76.9%) cases with a median time between MitraClip implantation and IE symptom onset of 5 [2–16] months. *Staphylococcus aureus* was the major causative microorganism (46%). Surgical mitral valve replacement was needed in 50% of patients. A conservative medical approach was considered in the remainder. The overall in-hospital mortality rate was 50% (surgical group: 38.4%; medical group: 58.3%; *p* = 0.433).

**Conclusion:**

Our results suggest that MitraClip-related IE affects elderly, comorbid patients, is mostly due to *Staphylococcus aureus*, and has a poor prognosis irrespective of the therapeutic approach. Clinicians must be aware of the features of this new entity among cardiovascular infections.

**Supplementary Information:**

The online version contains supplementary material available at 10.1007/s15010-023-02067-y.

## Introduction

In the past few decades, progress of interventional cardiology has boosted the development and widespread use of newer implantable cardiac devices, meant to correct structural or functional cardiac defects. Knowledge of devices and their infectious complications is a required skill for infectious disease physicians caring for these patients [1].

The MitraClip^©^ system (Abbott Vascular, Santa Clara, CA, USA) has been developed to correct severe functional (FMR) or degenerative mitral valve regurgitation (DMR), in patients deemed to be unfit for open-heart cardiac surgery [2–4]. MitraClip was first implanted in 2003 and obtained CE marking in Europe in 2008. The procedure essentially resembles the edge-to-edge mitral valve repair, but is performed percutaneously [5]. In the COAPT trial, MitraClip implantation reduced hospitalizations for heart failure when compared to optimal medical therapy alone (HR 0.53; 95% CI 0.4–0.7; *p* < 0.001) [4]. Therefore, the European Society of Cardiology suggests this procedure should be considered in carefully selected patients with DMR who are symptomatic despite optimal medical therapy and fulfill the COAPT trial inclusion criteria [6].

MitraClip is perceived to be less prone to infections compared to traditional surgical prostheses, but infective endocarditis (IE) following its implantation may occur and sparse data are currently available. The EVEREST II trial [2] reported an incidence of IE following MitraClip implantation of as low as 1.1% (2/184 pts) during the 12-months follow-up, and other studies suggested this risk to range between 0 and 1.3% [7]. However, the real impact of this complication might be larger, in light of growing procedure rates. Asmarats et al. [8] reported that most patients developing IE after transcatheter edge-to-edge mitral valve repair had a considerably high rate of comorbidities, showing a mean surgical risk, assessed by logistic EuroScore, of > 40%. Considering both the growing implantation rates and the common frailty of patients undergoing MitraClip implantation, it appears crucial to better characterize clinical features of MitraClip IE. This is particularly compelling in view of the absence of specific guidelines on the diagnosis and treatment of this condition.

In this systematic review, we aimed at collecting and summarizing current evidence to generate key information regarding clinical characteristics, management, and outcome of patients who developed IE on MitraClip devices.

## Methods

All procedures used in this systematic review were consistent with PRISMA guidelines (see Supplementary Material, Table S1).

### Selection criteria and case definition

For this systematic review, we included case series and case reports that described adult patients with MitraClip IE. We defined IE according to ESC 2015 criteria [9] and only patients fulfilling criteria for definite diagnosis were included. The device involvement was defined as evidence of vegetation on the clip or the mitral valve leaflets in carriers of the device, according to expert opinion [8], and similar to other cardiac implantable device infection criteria [10].

### Search strategy and data sources

We conducted a comprehensive literature search of PubMed, Google Scholar, Embase, and Scopus between January 2003 and March 2022. MitraClip was first implanted in 2003 [11] so we did not look for earlier reports. Study investigators (LB and MSR) designed the search strategy and conducted searches. We used controlled vocabulary along with keywords to search for studies including cases of MitraClip-associated IE. Full search strategies are provided in the supplementary material (Table S2). Two authors (LB and MSR) independently reviewed the titles and abstracts of the identified studies, and those not complying with the study inclusion criteria were excluded (Table 1). Reference lists of included studies were reviewed for relevant studies.

**Table 1:** Inclusion criteria for identified records

Criteria	Inclusion	Exclusion
(P) Population	Adult patients who underwent percutaneous transcatheter mitral valve repair with MitraClip system	Patients with open-heart edge-to-edge mitral valve repair or patients with transcatheter repair with devices other than MitraClip
(E) Exposure	Definite infective endocarditis diagnosed according to 2015 ESC criteria [9] with device involvement	Patients with infective endocarditis after MitraClip but not involving this device
(C) Comparator	Not applicable	Not applicable
(O) Outcome	Description of clinical characteristics, management, and outcomes	Not applicable

### Data items and collection process

Two investigators (LB and MSR) independently extracted data from included articles. For each study, the following variables were collected: year of publication, number of cases, baseline patient characteristics (age, sex, comorbidities, logistic EuroScore II at the time of MitraClip-related IE episode), residual mitral regurgitation after MitraClip implantation, presenting symptoms (including embolic phenomena), time between implantation and IE symptom onset, IE-related echocardiographic data (vegetation size and location), causative microorganism, type of treatment (medical vs surgical), in-hospital mortality, 1-month mortality, and reason for death.

**Fig. 1:**
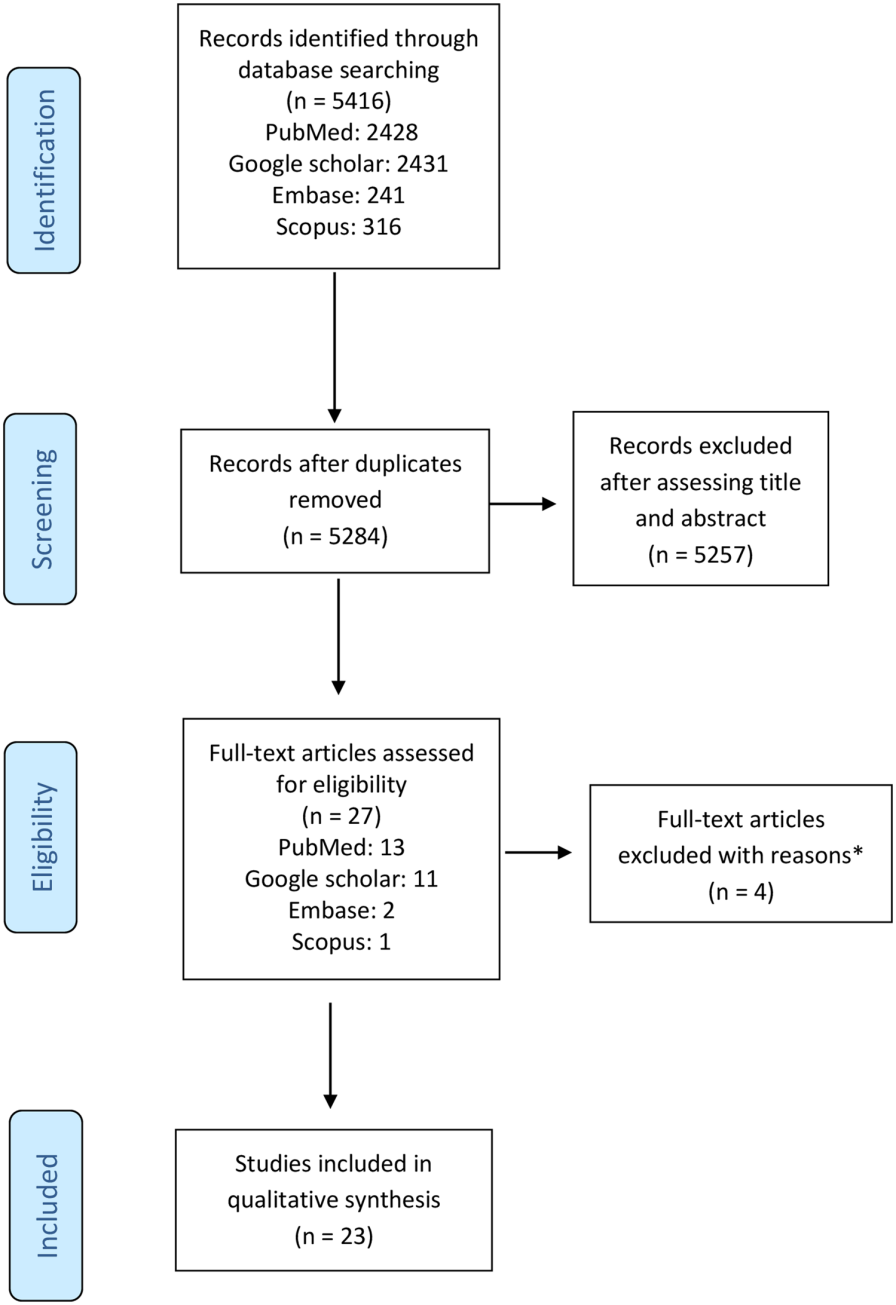
PRISMA study flow diagram. *The reasons for excluding four articles are: IE after open-heart edge-to-edge mitral valve repair = 1; IE on ‘Pascal ACE’ device = 1; Articles not found = 2

Two authors (LB and MSR) performed the study selection process according to pre-specified inclusion criteria (Table 1). Conflicts in data abstraction were resolved by consensus. The screening and selection process are presented in Fig. 1.

### Risk of bias

The Joanna Briggs Institute critical appraisal checklist was used to assess the quality of case reports (see Supplementary Material, Table S3) [12]. The Joanna Briggs Institute is an independent, international, not-for-profit research and development organization based in the Faculty of Health and Medical Sciences at the University of Adelaide, South Australia. Notwithstanding the strict methodology, potential bias of underestimation cannot be excluded.

### Analytical approach

Extracted variables that were continuous were presented as median and interquartile range [IQR], whereas categorical variables were presented as a proportion (observed cases/total number of cases). Analyses were performed using the statistical software for Windows Statistical Package for Social Sciences v. 22 (SPSS, Inc., Chicago, Illinois, USA). No meta-analysis was done.

## Results

### Results of the search

The database search identified 5416 records. After removing duplicates, we included in the screening phase, 5284 records. By assessing titles and abstracts, we excluded 5257 records. Twenty-seven records were assessed for eligibility and four full-text articles were excluded, leaving 23 records [13–35] included in the study for the qualitative synthesis (Fig. 1). The reasons for excluding four articles are detailed in Fig. 1 [36].

### Baseline patients’ characteristics

From the included studies, we collected data on 26 patients with IE following MitraClip implantation, mostly male (57.7%) with a median age of 76 [61–83] years. The cohort showed a high rate of comorbidities, which are displayed in Table 2, with a median logistic EuroScore/EuroScore II at the time of IE episode of 41%. A residual mitral regurgitation after MitraClip implantation and prior to the IE episode was present in 53.8% (14/26 pts) of reported cases, being mild in the majority (64.3%—9/14 pts) (Table 2).

**Table 2:** Baseline clinical characteristics of patients with MitraClip-related infective endocarditis

	MitraClip IE
Total number	26
Age (years), median [IQR]	76 [61–83]
Sex, number (%)	
Male	15 (57.7)
Female	9 (34.7)
Not available	2 (7.6)
Comorbidities, number (%)	
Ischemic heart disease	11 (42.3)
Systemic arterial hypertension	10 (38.4)
Chronic kidney disease	8 (30.7)
Chronic obstructive pulmonary disease	6 (23.1)
Diabetes	4 (15.4)
Residual mitral regurgitation (after MitraClip implantation), number (%)	
Mild	9 (34.6)
Moderate	3 (11.5)
Severe	2 (7.7)

### Clinical presentation of IE on MitraClip

A case-by-case detailed description is shown in Table 3. The most common symptoms at onset were fever (65.8%) and heart failure (42.3%), followed by systemic embolic phenomena in 15.3% of cases. Two cases presented with features of cardiogenic shock with refractory hypotension. Uncommon presentation as complete heart block was reported in one case. The IE episode had a rather early onset (< 12 months from implantation) in 20 (76.9%) cases with a median time between MitraClip implantation and IE symptom onset of 5 [2–16] months. We did not notice any difference in terms of causative microorganism and mortality when separating patients according to the time of IE onset (< 5 months or ≥ 5 months, data not shown).

**Table 3:** Clinical, microbiological, and echocardiographic data regarding the MitraClip-related infective endocarditis episode

	Symptoms	Emboli	Timing of symptoms onset (months)	Etiology	TEE performed	Vegetation	Vegetation size (mm)	Vegetation location
Case 1 (Kluge et al.) [13]	Fever, dyspnea	No	0.5	*S. aureus*	Yes	Yes	NA	Clip
Case 2 (Maznikoski et al.) [14]	Fever, dyspnea	No	1	NA	No	No	NA	NA
Case 3 (Vazir et al.) [15]	Sistemic emboli	Yes	14	*Streptococcus* spp.	No	Yes	10	Clip
Case 4 (Monsefi et al.) [16]	NA	NA	8	*S. aureus*	No	Yes	NA	Clip
Case 5 (Saito et al.) [17]	Dyspnea	No	36	CONS	No	Yes	15	Clip
Case 6 (Boeder et al.) [18]	Fever, hypotension	No	0.5	*S. aureus*	No	Yes	NA	NA
Case 7 (Boeder et al.) [18]	Fever	No	1	*S. aureus*	No	Yes	8	Clip
Case 8 (Russo et al.) [19]	Complete heart block	No	36	*S. aureus*	No	Yes	NA	Clip
Case 9 (Weiss et al.) [20]	Dyspnea	No	4	*E. faecalis*	No	Yes	12	Clip
Case 10 (Weiss et al.) [20]	Acute heart failure	No	2	*E. faecalis*	No	Yes	NA	Clip
Case 11 (Hermanns et al.) [21]	Fever, dyspnea	No	30	*S. aureus*	Yes	Yes	20	Leaflet
Case 12 (Hermanns et al.) [21]	Dyspnea	No	6	*S. aureus*	NA	NA	NA	NA
Case 13 (Rambhujun et al.) [22]	Fever, Fatigue	No	1	*S. aureus*	Yes	Yes	5	Clip
Case 14 (Kadoya et al.) [23]	Fever	No	2	*S. aureus*	Yes	Yes	NA	Clip
Case 15 (Leow et al.) [24]	Fever, dyspnea, confusion	No	2.5	*E. faecalis*	Yes	Yes	4	Leaflet
Case 16 (Nicolo et al.) [25]	Fever, cardiogenic shock	No	24	*B. haenselae*	No	Yes	NA	Leaflet
Case 17 (Papamanoli et al.) [26]	Fever	No	4	*S. oralis*	Yes	Yes	20	Clip
Case 18 (Perier et al.) [27]	Fever	No	1	CONS	No	Yes	9	Clip
Case 19 (Pudis et al.) [28]	Fever	Yes		*Abiotrophia* spp.	No	NA	NA	NA
Case 20 (Rempfer et al.) [29]	Heart failure, low back pain	No	48	*Corynebacterium* spp.	No	Yes	NA	Leaflet
Case 21 (Roslan et al.) [30]	Fever, dyspnea	No	0.2	*S. aureus*	Yes	Yes	NA	Clip
Case 22 (Shah et al.) [31]	Dyspnea	No	2	*S. aureus*	Yes	Yes	NA	Clip
Case 23 (Tayyar et al.) [32]	Fever, stroke	Yes	12	*P. aeruginosa*	Yes	Yes	20	Clip
Case 24 (Zapolski et al.) [33]	Fever, stroke	Yes	6	CONS	Yes	Yes	10	Leaflet
Case 25 (Hristakos et al.) [34]	Fever	No	3	*S. gordonii*	Yes	Yes	15	Leaflet
Case 26 (Frerker et al.) [35]	Fever, dyspnea	No	1	*S. aureus*	No	Yes	15	Clip

The most common causative pathogen of MitraClip was *Staphylococcus aureus*, which accounted for 46% of cases, followed by *Enterococcus faecalis* (11.5%), *coagulase-negative Staphylococci* (11.5%) and *Streptococcus *spp. (11.5%). Atypical microorganisms such as *Bartonella henselae*, *Abiotrophia *spp., *Corynebacterium *spp., and *Pseudomonas aeruginosa* were also reported.

According to echocardiographic data, the presence of a vegetation was reported overall in 23 patients with a median vegetation size of 12 [8.5–17.5] mm. However, transoesophageal echocardiography was performed in 11 (42.3%) cases only. In most cases (61.5%), the vegetation was located on the clip, whereas six patients (23%) showed native valve leaflet vegetation with apparent sparing of the device. In the remaining 15.3% of cases, information regarding vegetation location was not available.

### Treatment and outcome

**Fig. 2:**
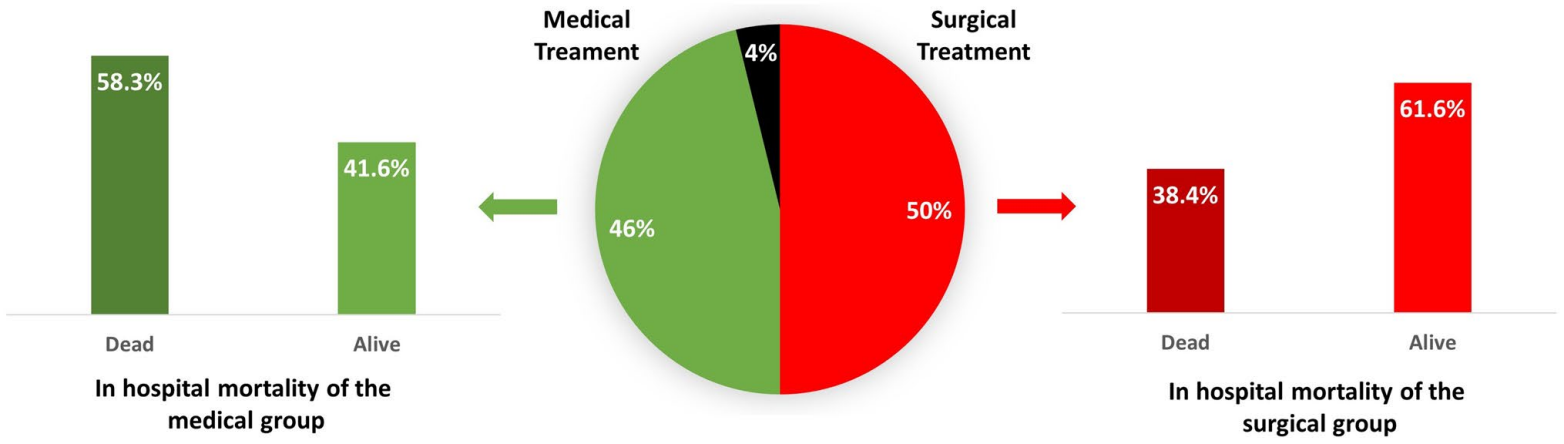
In-hospital mortality rates of MitraClip-related infective endocarditis according to the treatment choice. The central pie chart shows the percentage of patients treated surgically (red) and treated medically (green). Data not available in black. On the right and on the left, two bar charts show in-hospital mortality rates in the surgical (red) and in the medical group (green), respectively

A surgical approach with open-heart mitral valve replacement was the treatment performed in 13 (50%) patients, whereas the remainder (12 cases—46%) was managed only medically with systemic antimicrobial therapy. In one case [30], information regarding treatment and outcome was not available. The overall in -hospital mortality rate was very high, 50%. Mortality in the surgical group was 38.4%, whereas it was 58.3% in the medical group (*p* = 0.433, Fig. 2). Death was related to complications of IE in all cases. Similar mortality data were observed at 1 month after discharge (data not shown).

## Discussion

In this systematic review, we summarized the existing evidence in terms of clinical features and outcomes of MitraClip IE. Our results suggest this condition affects elderly, comorbid patients, is mostly due to *Staphylococcus aureus*, and has often a poor prognosis regardless of the therapeutic approach chosen (medical only vs medical and surgical).

Current studies report the median age of patients with IE to range between 60 and 68 years [37, 38]. In patients with MitraClip-related IE, the median age was substantially higher, consistent with this technique being usually applied to older patients with comorbidities deemed unfit for open-heart valve replacement [6]. Undoubtedly, this demographic feature has a major impact on the overall mortality of IE following MitraClip implantation, and clinicians called to manage this serious clinical condition should be aware of the frail conditions of affected patients. Older age also partially explains the high burden of comorbidities seen in these patients (Table 2).

The 2015 ESC guidelines for management of IE [9] report that up to 90% of patients with IE present with fever. In MitraClip IE, the prevalence of fever was in fact markedly lower (around 60%). This difference is consistent with prior data, showing older patients with IE are less prone to develop systemic signs and symptoms, including fever [39]. In contrast, as observed in other subsets of patients with native valve IE [8], heart failure was a common complication of MitraClip IE, related to both de novo valve dysfunction and sepsis-related cardiac dysfunction [40].

Interestingly, embolic complications occurred in a small share of MitraClip IE cases. The overall rate of embolic phenomena in IE ranged between 20 and 50% [9, 41, 42]. Moreover, previous studies underline that this risk is increased in patients with *S. aureus* etiology and vegetation size > 10 mm [43] and our data show that both of these features occur frequently in patients with MitraClip-related IE. The low rate of embolism in this setting could be related to advanced patient age [41] as well as use of anti-thrombotic medications after device implantation [44].

It was interesting to note that MitraClip-related IE presented in most cases within 1 year after implantation. This suggests the infection could be acquired perioperatively in a substantial percentage of cases. Moreover, previous studies showed MitraClip device stimulates a local histopathological healing response with a complete encasement in a collagen rich matrix within about 300 days [45, 46]. Likely, this process drastically reduces the risk for pathogen seeding, therefore making late MitraClip-related IE onset a less frequent phenomenon.

The best therapeutic approach of this condition is still debated and relies on a case-by-case evaluation, taking into account clinical conditions, hemodynamic stability, and preoperative risk. It should be underlined that no available guidelines or consensus paper on the management of MitraClip IE exist. This reflects the results of our study, in which almost 50% of cases were managed with systemic antimicrobials only, with the other half undergoing also open-heart cardiac surgery. Our data show that patients managed surgically had a lower mortality rate and this approach needs to be considered seriously despite of the extremely high preoperative risk. However, we did not observe any statistically significant difference in terms of mortality between the two groups, possibly due to the small sample size. Further studies are needed to understand the best therapeutic approach for these patients.

The in-hospital mortality rate of MitraClip-related IE is extremely high and mostly reflects that of prosthetic valve IE, ranging between 20 and 40% [9], consistent with advanced age and comorbid conditions.

In conclusion, IE is a severe complication of MitraClip implantation, and clinicians must be aware of the frail baseline clinical conditions of affected patients, who are often elderly and comorbid. We have provided data to help clinicians recognize and treat this condition.

### Supplementary Information

Below is the link to the electronic supplementary material.

Supplementary file1 (DOCX 38 KB)

## Data Availability

Not applicable.
